# Reestablishment of Ischemia-Reperfusion Liver Injury by N-Acetylcysteine Administration prior to a Preconditioning Iron Protocol

**DOI:** 10.1155/2013/607285

**Published:** 2013-10-27

**Authors:** Virginia Fernández, Romina Vargas, Valentina Castillo, Nicolás Cádiz, Daniela Bastías, Sebastián Román, Gladys Tapia, Luis A. Videla

**Affiliations:** Molecular and Clinical Pharmacology Program, Institute of Biomedical Sciences, Faculty of Medicine, University of Chile, Casilla 70000, Santiago 7, Chile

## Abstract

The role of iron (Fe)-induced prooxidant status in Fe preconditioning against ischemia (1 h)-reperfusion (20 h) induced liver injury was assessed using N-acetylcysteine (NAC) (1 g/kg) before Fe (50 mg/kg), given to male Sprague Dawley rats on alternate days during 10 days. IR significantly increased serum aspartate transaminase (AST) and alanine transaminase (ALT) levels, with drastic changes in liver histology, hepatic glutathione depletion, and nuclear factor-*κ*B (NF-*κ*B) p65 diminution (*P* < 0.05) (ELISA). Fe-induced liver oxidative stress, as evidenced by higher protein carbonyl/glutathione content ratios (*P* < 0.05) at days 11 and 12 after treatment, was abolished by NAC. Under these conditions, short-term Fe administration exerted significant protection against IR liver injury, as shown by 85% and 60% decreases in IR-induced serum AST and ALT (*P* < 0.05), respectively, and normalization of hepatic histology, glutathione levels, and NF-*κ*B activation, changes that were suppressed by NAC administration prior to Fe. Results of this study indicate that NAC administration prior to an iron protocol reestablishes IR liver injury, supporting the role of Fe-induced transient oxidative stress in hepatoprotection and its potential clinical application.

## 1. Introduction

Liver damage leading to cellular death is associated with a number of clinical settings underlying ischemia-reperfusion (IR) episodes, such as those occurring during organ procurement for transplantation, hepatic resection, low-blood pressure conditions, and abdominal surgery requiring hepatic vascular occlusion [1]. IR injury is a phenomenon in which cellular damage due to hypoxia is exacerbated upon restoration of O_2_ and nutrient supply. In fact, IR injury to the liver represents an important problem affecting transplantation outcome, leading to up to 10% of early organ failure, and increasing the incidence of both acute and chronic rejection [2]. Consequently, significant reduction or abrogation of the adverse effects of liver IR injury should increase the number of successful surgical procedures. 

In order to limit the detrimental effects of liver IR by enhancing the resistance of the organ, experimental hepatic preconditioning has been extensively explored in recent years, including the exposure of the liver to conditions triggering a mild oxidative stress status such as ischemic preconditioning [3]. In the latter strategy, the liver is submitted to a brief period of ischemia followed by a short period of reperfusion, previous to the prolonged ischemia. Ischemic preconditioning has been useful in human liver resections and in human liver transplantation; however, it remains controversial at present time [4]. For these reasons, our group has recently undertaken the evaluation of alternate experimental noninvasive liver preconditioning strategies that might have application in the clinical setting. In this respect, administration of thyroid hormone (T_3_) [5], n-3 long-chain polyunsaturated fatty acids (n-3 LCPUFA) [6], combined T_3_ and n-3 LCPUFA protocol [7], or iron (Fe) [8] have proved to be effective in the prevention of liver IR injury, conditions that underlie mild oxidative stress development.

As an essential micronutrient and biocatalyst of oxidoreduction processes involving reactive oxygen species (ROS) generation, Fe triggers cytoprotective responses at low levels. In the heart, Fe-induced protection is associated with upregulation of antioxidant enzymes [9, 10], a feature also observed in oligodendroglia cells [11]. Cell protection may be related to enhancement in the hepatic labile Fe pool, a compartment corresponding to a low-molecular-weight pool of weakly chelated Fe that readily passes through cells catalyzing ROS generation [12]. This is accomplished by Fe-catalyzed conversion of superoxide radical and hydrogen peroxide into hydroxyl radical, via the Fenton reaction or the Fe-assisted Haber-Weiss reaction, which triggers the generation of secondary bioradicals [12]. Consequently, Fe-induced redox signaling was proposed to underlie liver Fe preconditioning through activation of transcription factors Nrf2, STAT3, and NF-*κ*B triggering antioxidant and acute-phase responses [4]. In addition, upregulation of ferritin expression allowing sequestration of excess Fe is also achieved [8], an effect involving Fe-regulatory protein/Fe-responsive element posttranscriptional regulation [4]. This view is supported by reports showing that heart protection by ischemic preconditioning involves a pathway triggered by Fe and mediated by ferritin upregulation [13], whereas methemoglobin- or thrombin-dependent preconditioning against hemin toxicity in glial cells is blocked by desferioxamine or 5,5′-bipyridyl, Fe chelators that prevent ferritin induction [14]. In view of these considerations, this study was aimed at testing the hypothesis that Fe-induced liver preconditioning is triggered by the transient oxidative stress status associated with the Fe protocol. For this purpose, Fe liver preconditioning was assessed in a model of partial hepatic IR injury in the rat, either without or with pretreatment with the antioxidant N-acetylcysteine (NAC) [15], the results of which were correlated with parameters related to oxidative stress, and NF-*κ*B activity. 

## 2. Materials and Methods

### 2.1. Animal Preparation

**Figure 1:**
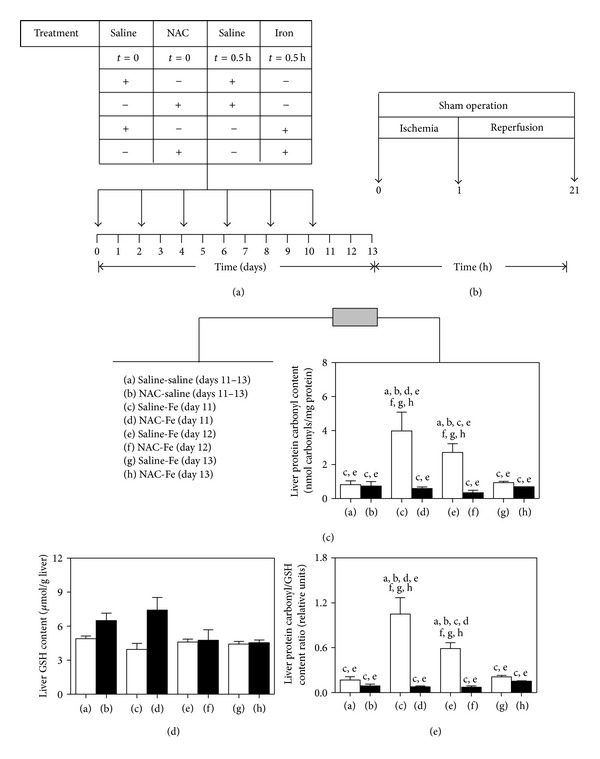
Experimental short-term procedure for N-acetylcysteine (NAC) and/or iron (Fe) administration in the rat (a), ischemia-reperfusion (IR) protocol (b), liver protein carbonyl content (c), liver reduced glutathione (GSH) content (d), and protein carbonyl/GSH content ratios (e). Animals were given either saline or NAC (1 g/kg) or saline followed by Fe (50 mg/kg) or saline, 0.5 h after NAC, at time 0 (controls), 2, 4, 6, 8, and 10 days. Liver oxidative stress-related parameters (protein carbonyl and GSH levels expressed as protein carbonyl/GSH content ratios) were determined at days 11, 12, and 13. At day 13, groups of animals were subjected to sham operation or 1 h ischemia followed by 20 h reperfusion. Blood and liver samples were obtained at the end of the reperfusion period (21 h) for assessment of serum transaminases, liver histology, and hepatic GSH content and p65 NF-*κ*B DNA binding. In (c), (d), and (e), data are expressed as means ± SEM for 3 to 11 animals per group. Significance studies (one-way ANOVA and Newman-Keuls' test; *P* < 0.05) are indicated by the letters identifying each experimental group.

Male Sprague Dawley rats (Bioterio Central, ICBM, Faculty of Medicine, University of Chile) weighing 140–160 g were housed on a 12-hour light/dark cycle and were provided with rat chow and water *ad libitum*. Intraperitoneal (ip) injections of N-acetylcysteine (NAC) and Fe-dextran, 0.5 h after NAC, were administered every second day over a 10 day period (Figure 1(a)). At time zero, animals received ip doses of either 1.0 g/kg NAC or saline, followed by ip doses of 50 mg of Fe-dextran/kg, or equivalent volumes of saline (controls) thus comprising four experimental groups, namely, saline-saline, NAC-saline, saline-Fe, and NAC-Fe (Figure 1(a)). The NAC dosage used (1 g/kg) is required considering the elimination half-life of 1 to 4.5 h reported for total NAC in rats [16]. The experimental short-term Fe administration protocol employed was previously shown to be devoid of liver toxicity and proinflammatory responses [8]. All ip injections were administered every second day over a 10 day period. Under these conditions each animal received 6 doses of either saline or NAC and Fe dextran or saline (Figure 1(a)). Liver reduced glutathione (GSH) [17], protein carbonyl levels (specific diagnostic kit, Cayman Chemical Company, Ann Arbor, MI, USA), and the respective protein carbonyl/GSH content ratios as oxidative stress-related parameters were assessed at days 11, 12, and 13 after treatment (Figure 1(a)). 

### 2.2. Model of Partial Ischemia-Reperfusion Injury

At day 13 after NAC plus Fe treatment (Figure 1(a)), rats were anesthetized with ip (1 mL/kg) zolazepam chlorhydrate (25 mg/mL) and tiletamine chlorhydrate (25 mg/mL) (Zoletil 50; Virbac S/A, Carros, France), and IR was induced by temporarily occluding the blood supply to the left and median lobes of the liver by means of a Schwartz clip (Fine Science Tools, Vancouver, BC, Canada) for 1 h followed by 20 h of reperfusion, as previously described [5]. The experimental design included saline and NAC plus Fe-treated rats subjected to either sham laparotomy or IR (Figure 1(b)), comprising eight experimental groups: (a) control-sham, (b) control-IR, (c) Fe-Sham, (d) Fe-IR, (e) NAC-sham, (f) NAC-IR, (g) NAC-Fe-sham, and (h) NAC-Fe-IR. At the end of the reperfusion period (Figure 1(c)), blood samples were obtained by cardiac puncture for serum aspartate transaminase (AST) and alanine transaminase (ALT) assessment (specific diagnostic kits; Biomerieux SA, Marcy l′ Etoile, France), and liver samples taken from the medial lobes were frozen in liquid nitrogen (NF-*κ*B assessment) or fixed in phosphate-buffered formalin, embedded in paraffin, and stained with hematoxylin-eosin (morphology assessment). In parallel groups, livers were perfused in situ with a cold solution containing 150 mM KCl and 5 mM Tris (pH 7.4) to remove blood, and samples taken from the medial lobes were used for evaluation of total GSH [17].

### 2.3. Assessment of the NF-*κ*B DNA Binding Activity

Nuclear protein extracts from liver samples were prepared with a nuclear extraction kit (Cayman Chemical Company, Ann Arbor, MI, USA), which allows separation of the cytoplasmic and nuclear fractions. Protein concentration in the nuclear fraction was determined with Bradford reagent at 590 nm. A nonradioactive assay kit (Cayman Chemical Company, Ann Arbor, MI, USA) was used for NF-*κ*B p65 DNA binding activity, which evaluates NF-*κ*B p65 binding to the response element by ELISA. The results are expressed as percentage of NF-*κ*B DNA binding activity in relation to a positive control (100%).

### 2.4. Ethics Statement

Experimental animal protocols and animal procedures complied with the Guide for the Care and Use of Laboratory Animals (National Academy of Sciences, NIH Publication 6–23, revised 1985) and were approved by the Bioethics Committee for Research in Animals, Faculty of Medicine, University of Chile (protocol CBA 0381 FMUCH). 

### 2.5. Statistical Analyses

Net changes in serum and liver parameters shown in the insets of figures were calculated by subtracting the mean values in the groups without IR (groups a, c, e, and g) from individual values in the respective groups subjected to IR (groups b, d, f, and h). Data showing Gaussian distribution according to the Kolmogorov-Smirnov test are expressed as the means ± standard error of the means (SEM) for the number of separate experiments indicated. One-way ANOVA (GraphPad Prism 4.0 software, GraphPad Software Inc. San Diego, USA) and Newman-Keuls^,^ test assessed the statistical significance of differences between mean values. A *P* value of less than 0.05 was considered significant.

## 3. Results

### 3.1. NAC Eliminates Fe-Induced Transient Oxidative Stress in Liver

The administration of 50 mg of Fe/kg (6 doses on alternate days) to fed animals (Figure 1(a)) significantly enhanced the protein carbonyl content of the liver by 385% and 230% at days 11 and 12 after treatment, respectively, without significant changes at day 13 (Figure 1(c)), compared to control values. Under these conditions, liver GSH levels were not modified by Fe (Figure 1(d)), thus leading to 518% and 247% increments (*P* < 0.05) in the respective protein carbonyl/GSH content ratios at days 11 and 12 after the Fe protocol compared to controls at days 11–13, without significant changes at day 13 (Figure 1(e)). The administration of 1 g of NAC/kg (6 doses on alternate days) (Figure 1(a)) abrogated the Fe-induced transient increase in the oxidative stress status of the liver, as shown by the significant reduction to control values of the enhanced protein carbonyl contents (Figure 1(c)) and the protein carbonyl/GSH content ratios observed at days 11 and 12 compared to those at day 13 of the experimental protocol (Figure 1(e)). 

### 3.2. NAC Abrogates Fe Liver Preconditioning

**Figure 2:**
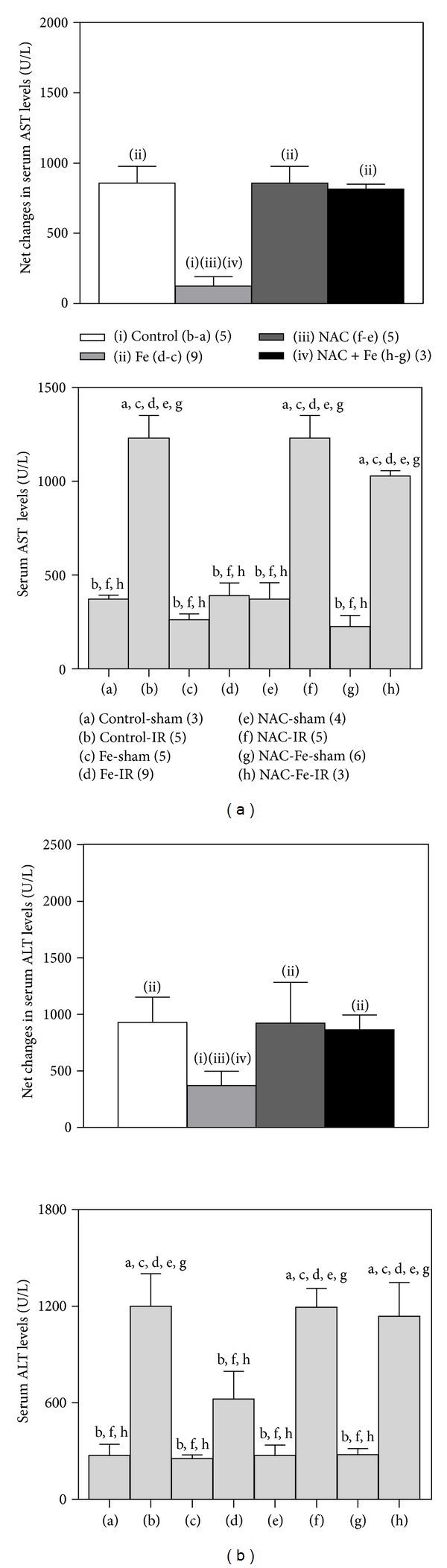
Effects of N-acetylcysteine (NAC) and/or iron (Fe) short-term administration on serum aspartate transaminase (AST) (a) and alanine transaminase (ALT) (b) levels in rats subjected to ischemia-reperfusion (IR). Animals were treated as shown in Figure 1. Data are expressed as means ± SEM for 3 to 9 animals per group. Significance studies (*P* < 0.05; one-way ANOVA and Newman-Keuls' test) are indicated by the letters identifying each experimental group. Inset: net changes in serum AST and ALT levels induced by IR correspond to the differences between groups (b-a) (controls), (d-c) (Fe), (f-e) (NAC), and (h-g) (NAC + Fe).

**Figure 3:**
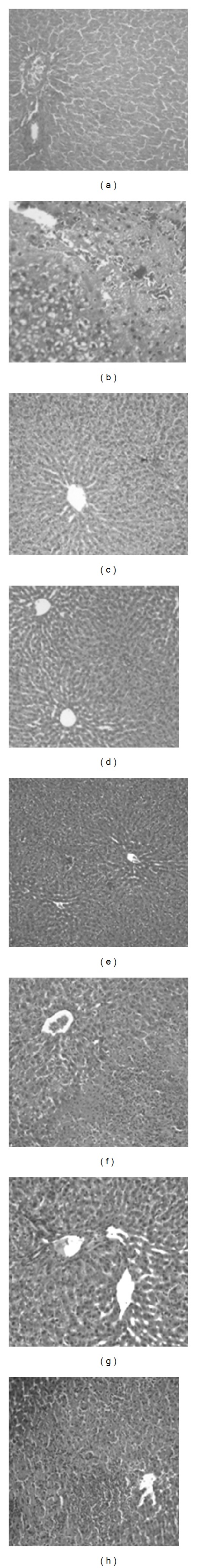
Effects of N-acetylcysteine (NAC) and/or iron (Fe) short-term administration on rat liver histology in animals subjected to ischemia-reperfusion (IR). Animals were treated as shown in Figure 1. Representative liver sections from control-sham (a), control-IR (b), Fe-sham (c), and Fe-IR (d) animals, and NAC-treated rats subjected to sham operation (e), IR (f), Fe-pretreatment and sham-operation (g), Fe pretreatment and IR (h) (hematoxylin-eosin-stained liver sections from a total of 3 to 4 animals per experimental group; original magnification ×20).

One hour of partial ischemia by vascular clamping of the portal triad followed by 20 h of reperfusion (Figure 1(b)) achieved extensive liver injury with minimal mortality, as shown by significant increments in serum AST (Figure 2(a)) and ALT (Figure 2(b)) levels (2.3- and 3.4-fold, resp.), in relation to sham-operated animals. In Fe-preconditioned animals both serum transaminases were similar to control sham operated animals (Figures 2(a) and 2(b)), with net diminutions of 85% and 60%, in relation to the unpreconditioned group (Figures 2(a) and 2(b), insets). After NAC administration, serum AST and ALT were significantly elevated by IR, in relation to the NAC-sham and NAC-Fe groups (Figures 2(a) and 2(b), resp.), thus showing abolishment of the Fe preconditioning effect and achieving net effects of IR similar to those of the unpreconditioned group (Figures 2(a) and 2(b), insets). In agreement with serum AST and ALT data, morphological characteristics of the liver assessed by light microscopy showed normal morphology in control-sham (Figure 3(a)) and Fe-sham (Figure 3(c)) animals, whereas control-IR rats exhibited distorted architecture with extensive centrolobular and peripheral necrosis and neutrophil infiltration (Figure 3(b)), changes that were not observed in Fe-IR rats (Figure 3(d)). In addition, control animals subjected to NAC-sham or to NAC-Fe sham conditions exhibited normal liver histology (Figures 3(e) and 3(g)), whereas the NAC-IR group showed mild to moderate congestion, scattered cellular necrosis (coagulation), and isolated sinusoidal neutrophil infiltration (Figure 3(f)). Similar liver histology was observed in the NAC-Fe-IR group (Figure 3(h)), supporting the suppression of Fe liver preconditioning against IR injury by NAC pretreatment. 

### 3.3. NAC Annuls Fe-Dependent Recovery of Total Liver GSH Content

**Figure 4:**
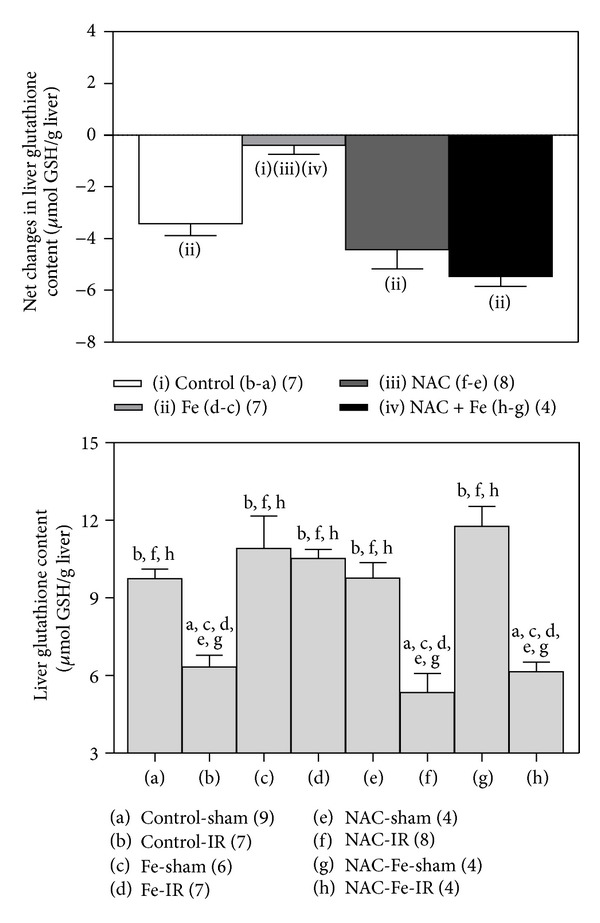
Effects of N-acetylcysteine (NAC) and/or iron (Fe) short-term administration on liver reduced glutathione (GSH) content in rats subjected to ischemia-reperfusion (IR). Animals were treated as shown in Figure 1. Data are expressed as means ± SEM for 4 to 9 animals per group. Significance studies (*P* < 0.05; one-way ANOVA and Newman-Keuls′ test) are indicated by the letters identifying each experimental group. Inset: net changes in liver GSH levels induced by IR correspond to the differences between groups (b-a) (controls), (d-c) (Fe), (f-e) (NAC), and (h-g) (NAC + Fe).

IR liver injury was found concomitant with a 35% depletion in liver GSH content over control values in unpreconditioned rats (Figure 4), with a net decrease of 3.42 ±0.46 (*n* = 7) *μ*mol/g liver (Figure 4, inset), an effect that was abolished by Fe preconditioning (Figure 4). A 45% reduction in GSH was observed in NAC-IR group compared to NAC-sham animals (net decrease of 4.44 ± 0.74 (*n* = 8) *μ*mol/g liver), which is comparable to that found upon NAC + Fe pretreatment in animals subjected to IR (48% decrease; net diminution of 5.62 ± 0.38 *μ*mol/g liver (*n* = 4) (Figure 4, inset). These data show that Fe-dependent recovery of the GSH content, induced by IR in the liver, is suppressed by NAC administration before Fe (Figure 4).

### 3.4. NAC Suppresses the Normalization of Hepatic NF-*κ*B DNA Binding Activity Induced by Fe

**Figure 5:**
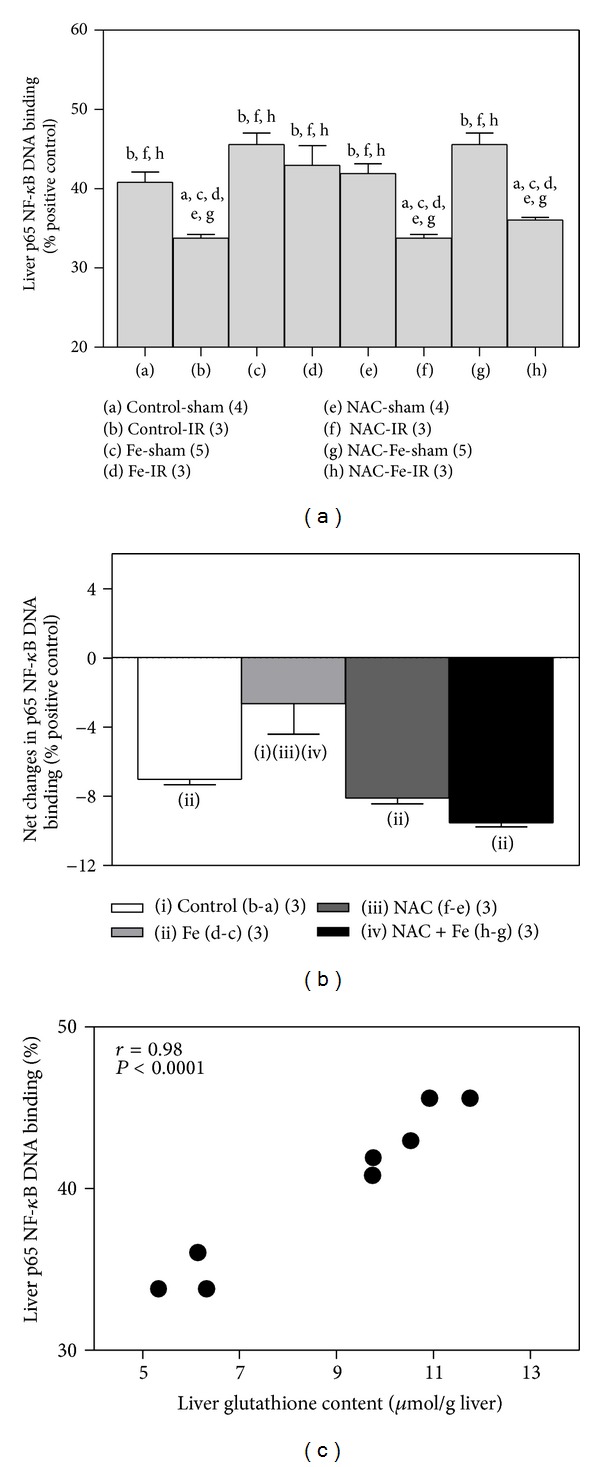
Effects of N-acetylcysteine (NAC) and/or iron (Fe) short-term administration on liver p65 nuclear factor-*κ*B (NF-*κ*B) DNA binding in rats subjected to ischemia-reperfusion (IR) (a), net changes in NF-*κ*B activation (b), and correlation between NF-*κ*B DNA binding and glutathione levels (c). Animals were treated as shown in Figure 1. Data are expressed as means ± SEM for 3 to 5 animals per group. Significance studies (*P* < 0.05; one-way ANOVA and the Newman-Keuls' test) are indicated by the letters (a) or numbers (b) identifying each experimental group. In b, net changes in liver p65 NF-*κ*B DNA binding induced by IR correspond to the differences between groups (b-a) (controls), (d-c) (Fe), (f-e) (NAC), and (h-g) (NAC + Fe).

IR led to a significant 17% diminution of liver NF-*κ*B p65 DNA binding activity, compared with control-sham-operated rats (Figure 5(a)), with a net decrease of 7.0 ± 0.3 arbitrary units (*n* = 3) (Figure 5(b)). Total recovery in the IR induced loss in hepatic NF-*κ*B activity was achieved by Fe preconditioning (Figure 5(a)), an effect that was not observed either after NAC (19% diminution; net decrease of 8.1 ± 0.3 (*n* = 3) arbitrary units) or NAC plus Fe (21% reduction; net decrease of 9.5 ± 0.2 (*n* = 3) arbitrary units) (Figure 5(b)). These data show that the Fe-dependent recovery of the diminished NF-*κ*B DNA binding activity by IR is wholly suppressed by NAC pretreatment, in concomitance with the elimination of the Fe-induced normalization of hepatic glutathione levels (Figure 4). Under the experimental conditions employed, NF-*κ*B activation exhibits a significant correlation with the glutathione content (*r* = 0.98; *P* < 0.0001) (Figure 5(c)), as indicator of the oxidative stress status of the liver. 

## 4. Discussion

Data presented in this work confirm the previous finding that 1 h of partial ischemia followed by 20 h reperfusion triggers substantial liver injury, with significant alteration of oxidative stress-related parameters and reduction in DNA binding of NF-*κ*B [5, 8]. Subchronic Fe administration led to transient oxidative stress development in the liver, an effect that is related to regulatory and protective actions and considered as a hormetic stimulus leading to preconditioning effects against IR [18]. Regulatory and protective effects of reversible oxidative stress on rat liver have been recently associated with transient ischemia [19] and the *in vivo* administration of thyroid hormone [5, 20] or n-3 LCPUFA [6] preconditioning strategies, constituting an important issue in redox-modulated cell signaling [21]. Development of transient liver oxidative stress in the time-interval of 24 to 72 h after short-term Fe administration (days 11 to 13 in the experimental protocol; Figure 1(a)) is characterized by (i) its association with enhancement in the hepatic labile Fe pool as a triggering mechanism [8], (ii) the lack of hepatotoxicity induction, and (iii) the substantial protection against IR liver injury. 

The present study demonstrates that Fe-induced transient oxidative stress has a causal role in liver preconditioning against IR injury, as shown by the use of NAC previous to each Fe dose. The NAC protocol employed leads to rapid and significant increases in NAC circulating levels, without detectable concentrations of the antioxidant 48 h after NAC administration and with comparable levels of hepatic GSH content in animals treated with NAC and control rats [20], thus assuring lack of interference of NAC with the oxidative stress response induced by IR. Under these conditions, NAC abrogated the transient oxidative stress produced by Fe, as evidenced by the normalization of liver protein carbonyl/GSH content ratio, and antagonized Fe-induced liver preconditioning against IR injury, as shown by the persistence of liver injury, GSH depletion, and reduction in NF-*κ*B DNA binding activity elicited by IR, which are suppressed by Fe in the absence of NAC treatment. Taking into account the antioxidant properties of NAC, namely, direct free radical scavenging action and stimulation of GSH biosynthesis [15], abolishment of the oxidative stress component induced by Fe may be responsible for NAC-dependent suppression of Fe preconditioning. Furthermore, abrogation of Fe preconditioning by NAC may also involve maintenance of relevant sulfhydryl groups of cellular proteins in the reduced state [22, 23], leading to modifications of the signal-transduction pathways underlying liver protection by Fe. 

In conclusion, data presented in this study indicate that Fe-induced reversible oxidative stress in the liver is essential for protection against IR injury, as evidenced by the reestablishment of liver damage after the administration of NAC before Fe. Loss of Fe preconditioning by NAC is associated with failure of two relevant aspects involved in liver cytoprotection, namely, the mild prooxidant status of the liver developed and the recovery of NF-*κ*B DNA binding activity. This contention is supported by the significant correlation established between NF-*κ*B DNA binding levels and the glutathione status of the liver. Under the IR conditions used, reduction of liver nuclear NF-*κ*B DNA binding with loss of its signaling functions leading to cytoprotection may contribute to liver injury [4, 24]. In agreement with these views, recent studies by our group revealed that short-term Fe administration activates redox-sensitive transcription factor Nrf2 also involved in cytoprotection [25], an effect that is restrained by NAC in the case of T_3_ upregulation [26]. Nrf2 is known to trigger the expression of antioxidant proteins, in addition to phase-II detoxification enzymes and phase-III transporters of the xenobiotic biotransformation pathway [27, 28]. The results of this study support Fe preconditioning as a novel strategy to protect the liver and other organs against IR injury, thus constituting an alternate preconditioning strategy with potential clinical application. In fact, under controlled Fe protocols in low-dose ranges hepatotoxicity is not observed (Figure 3) [8] and minimal side effects to extrahepatic tissues are reported [12, 29]. Moreover, repeated intramuscular or intravenous injections of 100–125 mg/day of Fe complexes 1 to 3 times/week for 4 to 12 weeks are a well-tolerated therapeutic strategy in the treatment of human anemia in Fe deficiency [30, 31].
